# The Dual Role of STAT1 in Ovarian Cancer: Insight Into Molecular Mechanisms and Application Potentials

**DOI:** 10.3389/fcell.2021.636595

**Published:** 2021-03-23

**Authors:** Xin Li, Fanchen Wang, Xiaolin Xu, Jinguo Zhang, Guoxiong Xu

**Affiliations:** ^1^Research Center for Clinical Medicine, Jinshan Hospital, Fudan University, Shanghai, China; ^2^Department of Oncology, Shanghai Medical College, Fudan University, Shanghai, China

**Keywords:** chemoresistance, immune response, microenvironment, tumorigenesis, signaling pathway, stemness

## Abstract

The signal transducer and activator of transcription 1 (STAT1) is a transducer protein and acts as a transcription factor but its role in ovarian cancer (OC) is not completely understood. Practically, there are two-faced effects of STAT1 on tumorigenesis in different kinds of cancers. Existing evidence reveals that STAT1 has both tumor-suppressing and tumor-promoting functions involved in angiogenesis, cell proliferation, migration, invasion, apoptosis, drug resistance, stemness, and immune responses mainly through interacting and regulating target genes at multiple levels. The canonical STAT1 signaling pathway shows that STAT1 is phosphorylated and activated by the receptor-activated kinases such as Janus kinase in response to interferon stimulation. The STAT1 signaling can also be crosstalk with other signaling such as transforming growth factor-β signaling involved in cancer cell behavior. OC is often diagnosed at an advanced stage due to symptomless or atypical symptoms and the lack of effective detection at an early stage. Furthermore, patients with OC often develop chemoresistance and recurrence. This review focuses on the multi-faced role of STAT1 and highlights the molecular mechanisms and biological functions of STAT1 in OC.

## Introduction

The signal transducer and activator of transcription (STAT) protein is an essential component of the interferon (IFN)/Janus kinase (JAK) signaling. There are at least seven members in the STAT family, including STAT1, 2, 3, 4, 5A, 5B, and 6, involved in immune surveillance, defense, and homeostasis (Ihle, 2001; O’Shea et al., 2002). STAT1, also known as STAT91, is the first discovered member of the STAT family but it is less understood in tumorigenesis compared to STAT3, a most well-studied member in this family. The gene of *STAT1* is located in 2q32.2 and contains 45,215 bp (Yamamoto et al., 1997). STAT1 protein generally exists as an inactive form in the cytoplasm (Zhang and Liu, 2017). In the canonical signaling pathway, STAT1 is initially phosphorylated and activated by the receptor-activated kinases such as JAK in response to IFN stimulation (Ihle and Kerr, 1995). The activated STAT1 forms homodimer or heterodimers with other STATs and translocates from the cytosol to the nucleus where it acts as a transcription factor to regulate its target genes.

Ovarian cancer (OC) is the most lethal gynecological disease in women worldwide. In the United States, 21,750 new-diagnosed cases and 13,940 death cases are estimated to occur in 2020 (Siegel et al., 2020). Because of symptomless or atypical symptoms at the early stage, patients with OC are often diagnosed at an advanced stage due to the lack of effective detection at an early stage, chemoresistance, and even recurrence (Torre et al., 2018). Although the mortality of OC patients keeps a stable or trend down for decades (Malvezzi et al., 2016), the prognosis of OC is still not significantly improved. Therefore, increasing the efficiency of prevention, early diagnosis, and more effective treatment for OC patients is most important (Natanzon et al., 2018; Walker and Sobel, 2018).

The mechanisms of OC development can generally be interpreted by the hallmarks of cancer (Hanahan and Weinberg, 2011), regarding the incidence, progression, recurrence, and drug-resistance (Matulonis, 2018). Hence, most therapeutic drugs are designed to target those molecules involved in tumorigenesis and tumor development (Hainaut and Plymoth, 2013). For example, many inhibitors of poly (ADP-ribose) polymerase (PARP), angiogenesis, RAS/RAF/MEK pathway, PI3K/AKT pathway, and immune checkpoints are currently available to treat patients with OC (Guan and Lu, 2018). In addition to these well-studied pathways and cellular processes, some molecules also contribute to OC progression, including ATP-binding cassette (ABC) transporters, astrocyte-elevated gene-1 (AEG1), BRCA1, BRCA2, CCNG1, C-X-C motif receptor 2 (CXCR2), IGF1R, p53, STAT1, and STAT3 (Blanco et al., 2010; Devapatla et al., 2015; Ween et al., 2015; Zhang and Zou, 2015; Zhou et al., 2015; Liu et al., 2018; Xu et al., 2019; Liang et al., 2020). Most of them are potential therapeutic targets but have not been developed as drugs tested in the clinical trial. Among different molecules indicated, STAT1 has emerged recently as an important STAT. Existing evidence reveals that STAT1 has both tumor-suppressing and tumor-promoting functions involved in angiogenesis, cell proliferation, migration, invasion, apoptosis, drug resistance, stemness, and immune responses mainly through interacting and regulating target genes at multiple levels. More and more studies of STAT1 in cancer unveil the mechanisms of tumor development and focus on the target of the immune response. However, STAT1 has two-faced effects on tumorigenesis and this dual role of STAT1 in OC is not completely understood (Zhang and Liu, 2017).

## Structure and Biogenesis of STAT1

**FIGURE 1:**
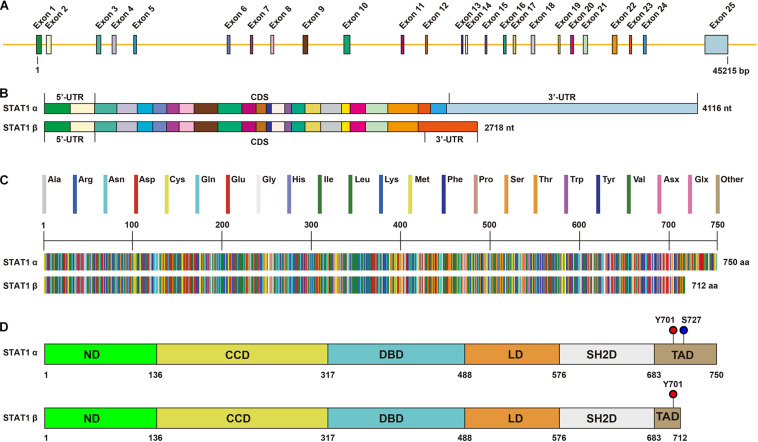
Structure of STAT1. **(A)** Genomic illustration of STAT1. The *STAT1* gene is located in 2q32.2 containing 45,215 bp. There are 25 exons in STAT1α and 23 exons in STAT1β. **(B)** Transcripts of STAT1α and STAT1β. The mRNA of STAT1α and STAT1β contains 4116 and 2718 bp, respectively. **(C)** Alignment of protein sequences of STAT1α (750 amino acids) and STAT1β (712 amino acids). **(D)** Illustration of the functional domains of STAT1. Phosphorylation sites of residues tyrosine 701 (Y701) and serine 727 (S272) are shown. ND, N-terminal domain; CCD, coiled-coil domain; DBD, DNA binding domain; LD, linker domain; SH2D, Src homology-2 domain; TAD, transactivation domain.

The gene of *STAT1* contains 25 exons (Figure 1A). Because of alternative splicing (Schindler et al., 1992), STAT1 has two transcripts: a full-length STAT1α and a truncated STAT1β (Figure 1B). STAT1α is the main form that has 750 amino acids, whereas STAT1β is a short form that has 712 amino acids (Figure 1C). STAT1α has two important phosphorylation sites at residues tyrosine 701 (Y701) and serine 727 (S727) located at the C-terminal transactivation domain (TAD) (Figure 1D), whereas STAT1β lacks part of the TAD but can be efficiently phosphorylated on Y701 (Oda et al., 2015).

The structure of STAT1 protein generally consists of the N-terminal domain (ND), coiled-coil domain (CCD), DNA binding domain (DBD), linker domain (LD), Src homology-2 domain (SH2D), and TAD. From N-terminus to C-terminus, each domain has its own function. The ND is implicated in the protein-protein interaction, nuclear translocation, and deactivation of STAT1 (Morris et al., 2018). The CCD functions as a scaffold for protein-protein interaction which is cooperated with other transcription factors and co-factors (Chen et al., 1998). The DBD is the direct binding site of DNA which helps STAT1 translocation from the cytosol to the nucleus. The LD is involved in regulating transcriptional activity (Mertens et al., 2015). The SH2D is the most conserved region that specifically binds to the phosphorylated-tyrosine site of receptors and is responsible for the dimerization and maintenance of the DNA-binding stability, whereas the TAD is the transactivation site activated after Y701 and/or S727 phosphorylation (Chen et al., 1998).

## Signaling Pathway and Biological Function of STAT1

**FIGURE 2:**
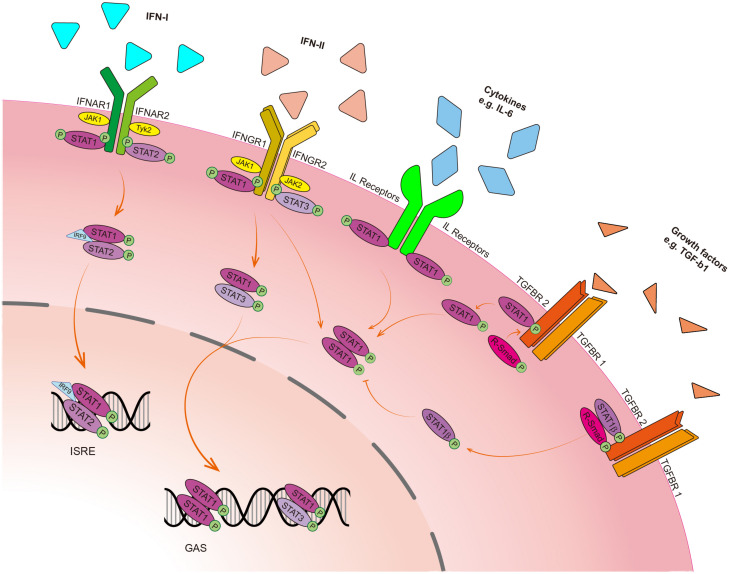
Schematic presentation of the STAT1 signaling pathway. In response to type I IFN (IFN-α and IFN-β), phosphorylated STAT1 binds to STAT2 and forms a heterodimer, which further forms a complex with IFN regulatory factor 9 (IRF9), along with interferon-stimulated gene factor-3 (ISGF-3), and then translocate into the nucleus to bind to the IFN-stimulated response element (ISRE) of target genes to regulate their transcription. In response to type II IFN (IFN-γ), phosphorylated STAT1 forms homodimer with STAT1 or heterodimer with STAT3. These dimers then translocate into the nucleus to bind to the gamma-IFN activation site (GAS) to perform the transcriptional activity. Cytokines such as interleukins (ILs) that bind to their receptors without the help of JAK can activate STAT1 and form STAT1 homodimer, followed by the nucleus translocation. In the crosstalk with the TGF-β signaling pathway, STAT1 constitutively interacts with TGF-β1 receptor. Upon TGF-β1 binding to its receptors (TGFBR1 and TGFBR2), the receptor-complex increases phosphorylation STAT1α on S727. Then, the activated STAT1α dissociates itself from the receptor complex and translocates into the nucleus. STAT1β blocks STAT1α homodimer formation.

Signal transducer and activator of transcription 1 can not only be activated by type I and type II IFNs in the canonical JAK/STAT1 signaling pathway but also be activated by other cytokines such as interleukins (e.g., IL-6) and growth factors [e.g., transforming growth factor-β1 (TGF-β1)] through the phosphorylation of Y701 residue in different patterns (Figure 2). As a transducer, STAT1 protein can transduce extracellular signals through transmembrane receptors. Interferon-α receptor 1 (IFNAR1) and IFNAR2 are receptors of type I IFN in response to IFN-α/-β, whereas interferon-γ receptor 1 (IFNGR1) and IFNGR2 are receptors of type II IFN in response to IFN-γ. Despite the confluence of the signals upon ligands binding to their receptors, STAT1 has distinct effects on targeted genes in different cells. The binding of IFNs to their receptors leads to the activation of receptor-associated JAK, which phosphorylates the tyrosine residue of receptors (Stark and Darnell, 2012). The phosphorylated receptors and activated JAKs recruit STAT1 by binding to the SH2D of STAT1 and phosphorylate STAT1 at Y701 residue, leading to the release of STAT1 from receptors and triggering the formation of STAT1/STAT1 homodimer, STAT1/STAT2 or STAT1/STAT3 heterodimers, and in turn, these dimers translocate from the cytosol to the nucleus where they bind to DNA binding sites of IFN-stimulated genes (ISG) (Shuai, 1994). STAT1/STAT2 heterodimer and IFN regulatory factor (IRF) 9 form a complex with ISG factor-3 (ISGF-3) (Ramana et al., 2000), which binds to the IFN-stimulated response element (ISRE) in the promoter of a target gene in response to IFN-α and IFN-β stimulation (Horvath, 2004). However, STAT1 homodimer and STAT1/STAT3 heterodimer bind to the gamma-IFN activation site (GAS), a chromatin open site in the promoter of a target gene in response to IFN-γ stimulation (Figure 2; Ramana et al., 2002).

The C-terminal TAD has a transactivation function and can recruit transcriptional co-factors to the promoter of IFN-γ-activated genes. For instance, the TAD of STAT1α recruits RNA polymerase II (RNA pol II) to IFN-induced guanylate-binding protein 2 (GBP2) and core Mediator complex to the GAS of the IRF1 and IRF8 promoters (Parrini et al., 2018). The TAD also functions to interact with the histone acetyltransferase CBP/p300 that promotes the DNA binding activity of STAT1 (Wojciak et al., 2009). The phosphorylation of STAT1α at S727 acquires a transactivation function (Chen et al., 1998). Cyclin-dependent kinase 8 (CDK8) has been reported to activate STAT1 by phosphorylation at S727, whereas a CDK8-specific-inhibitor can suppress STAT1 phosphorylation at S727 (Bancerek et al., 2013; Nitulescu et al., 2017). It seems that STAT1β has no transactivational function because of the absence of S727. However, despite lacking the TAD, STAT1β should not be considered as transactivation null. Studies using gene-modified mice suggest that the depletion of the TAD does not eliminate transcriptional activation in responses to IFN-γ signal. The conversion of S727 to alanine (S727A) results in a similar outcome (Semper et al., 2014), indicating that the function of the TAD does not rely on the phosphorylation of S727 alone. Furthermore, Y701 and S727 are two phosphorylated sites of STAT1 which may confer different functions of STAT1 (Nitulescu et al., 2017). Therefore, it is speculated that the ratio of STAT1α/STAT1β may govern the effects of STAT1 in tumorigenesis.

## Role of STAT1 in Ovarian Cancer

Signal transducer and activator of transcription 1 is overexpressed in OC compared with normal ovarian tissue (Tian et al., 2018; Liu et al., 2020) and is associated with the overall survival of patients (Josahkian et al., 2018). High levels of STAT1 evaluated by immunohistochemistry is associated with longer overall survival and progression-free survival of patients with high-grade serous ovarian cancer (HGSOC) (Table 1; Koti et al., 2015; Au et al., 2016; Josahkian et al., 2018). As a transcription factor and transducer protein, STAT1 can regulate its target genes and can be a target to be regulated by other molecules (Tables 2, 3). These STAT1-related regulators and mediators as well as STAT1 itself are highly important and have played roles in OC development and treatment outcome. However, the molecular mechanisms of the cellular processes underlying such regulations are complex and still not completely understood yet.

**TABLE 1:** High-level STAT1 associated with a better outcome of survival in patients with high-grade serous ovarian cancer.

Sample #	Region	Method	Outcome	References
65	Brazil	IHC	Longer DFS (*p* = 0.0358, HR = 0.432) Longer OS (*p* = 0.0469, HR = 0.329)	Josahkian et al., 2018
550	Canada	IHC	Longer PFS (*p* = 0.017, no HR data)	Au et al., 2016
183	Canada	IHC	Longer PFS (*p* = 0.02, HR = 0.73)	Koti et al., 2015

*DFS, disease-free survival; HR, hazard ratio; IHC, immunohistochemistry; OS, overall survival; PFS, progression-free survival.*

**TABLE 2:** Target genes regulated by STAT1 with biological function in ovarian cancer cells.

Gene	Regulation	Cell type	Biological function	References
CXCL10	Up (FC: >10)	SKOV3; OV-MZ-6; Human HGSOC cells; Mouse OC cells	Angiogenesis; Apoptosis; Proliferation; Migration; Th1 immune response	Au et al., 2017
GPI	Up	SKOV3; A2780	Apoptosis; Glycolysis; Proliferation	Rupaimoole et al., 2015
IDO	Up	A2774; OC316	Immune escape	Carbotti et al., 2015
IFI16	Up (FC: >60)	UWB1.289	N/A	Cardenas et al., 2019
IL-18BP	Up	SKOV3; A2780; A2774; OVCAR5	Immune suppression	Carbotti et al., 2013
iNOS	Up	ES-2	Angiogenesis	Trinh et al., 2015
CD44/CD133/NANOG/OCT4	Down	OVCAR3; OV3R-PTX	Stemness	Wang et al., 2020
Smad2	Down	SKOV-3; OVCAR3	TGF-β1 signaling	Tian et al., 2018
TNFSF15	Down	OVCAR3	Angiogenesis	Lu et al., 2014
xCT	Down	A2780; human primary OC cells	Cisplatin efflux; Platinum resistance	Wang et al., 2016

*Up, gene upregulated by STAT1; Down, gene downregulated by STAT1; FC, fold change of genes between STAT1 high and low cells; OC, ovarian cancer; EOC, epithelial ovarian cancer; HGSOC, high-grade serous OC; N/A, not available.*

**TABLE 3:** Summary of molecules that regulate STAT1 with biological function and chemo-responsiveness in ovarian cancer cells.

Molecule	STAT1 expression	Cell	Biological function	Chemo- responsiveness	References
DLX4	Up	ES-2	Angiogenesis	N/A	Trinh et al., 2015
HDAC4	Up	Human primary OC cells	N/A	Increase platinum resistance	Stronach et al., 2011
ITGB1	Up	HO-8910; HO-8910PM	Apoptosis; Invasion; CAM-DR	Decreases bevacizumab sensitivity	Zhang and Zou, 2015
TGF-β1	Up (pSTAT1-Y701); Down (pSTAT1-S727)	SKOV3; OVCAR3	Proliferation, migration and invasion	N/A	Tian et al., 2018

*Up, upregulation of STAT1; down, downregulation of STAT1; CAM-DR, cell adhesion mediated drug resistance; OC, ovarian cancer; pSTAT1, phosphorylated STAT1; N/A, not available.*

### Proliferation and Apoptosis

It has been reported that STAT1 promotes OC cell proliferation and suppresses apoptosis by up-regulating the expression of inducible nitric oxide synthase (iNOS) in OC tissues compared with normal ovarian tissues (Burke et al., 2017). Distal-less homeobox 4 (DLX4), which is overexpressed in OC and is associated with poor prognosis of patients with OC, can directly interact with STAT1 and activate STAT1, thus triggering the expression of iNOS (Trinh et al., 2015; Kielbik et al., 2019). Furthermore, DLX4-induced iNOS expression is eliminated in STAT1 loss-of-function mutation cells (Wen et al., 1995), indicating that STAT1 is a potential transactivator of iNOS. There is evidence that STAT1α can bind to the GAS of iNOS promoter, which is essential for the transcription of iNOS (Gao et al., 1997), leading to the generation of cytotoxic nitric oxide (NO) (Kielbik et al., 2019), and in turn, promoting OC progression (Li et al., 2017). On the other hand, iNOS-induced NO can promote apoptosis by increasing the expression of p53, a well-known tumor suppression gene (Leung et al., 2008). It has been demonstrated that integrin β 1 (ITGB1), which is upregulated in OC, promotes ovarian tumor growth and progression (Yang et al., 2014) and inhibits apoptosis by upregulating STAT1 expression. Silencing ITGB1 enhances the effect of bevacizumab treatment through inhibiting STAT1 (Zhang and Zou, 2015).

### Invasion and Metastasis

Signal transducer and activator of transcription 1 has been identified to promote cell adhesion, invasion, and migration in a variety of cancers (Greenwood et al., 2012) supported by most studies using adhesion assay, wound healing assay, transwell assay, and Western blot analyses to detect invasion markers such as matrix metallopeptidase 2 (MMP-2) and MMP-9 (Zhang and Zou, 2015). However, the detailed molecular mechanisms underlying STAT1-induced invasion and migration in OC remain unclear. As previously mentioned that ITGB1 is upregulated in OC and promotes ovarian tumor growth and progression (Yang et al., 2014), ITGB1 can upregulate STAT1 through activating focal adhesion kinase (FAK), the downstream kinase of ITGB1, leading to the upregulation of MMP-2 and MMP-9. Besides, STAT1 induces OC cell invasion and migration by suppressing the TGF-β signaling pathway (Tian et al., 2018).

Paradoxically, STAT1 has also been reported to inhibit metastasis of OC. Enhanced transcriptional levels of chemokine C-X-C motif ligand (CXCL) 9 (CXCL9), CXCL10, and CXCL11 are found in STAT1-overexpressed OC cells and may be related to longer overall survival and progression-free survival (Groom and Luster, 2011; Bronger et al., 2016; Au et al., 2017). Apart from that, a study using the OC ID8 cell inoculation mouse model further indicates that CXCL10 reduces ascites formation and tumor burden (Au et al., 2017). Another study using a murine cancer model demonstrates that the release of CXCL9/10 hampers tumor growth and metastasis (Kundu et al., 2005).

### Angiogenesis

Signal transducer and activator of transcription 1 has been reported to be a key regulatory factor of angiogenesis (Hsu et al., 2017) that is one of the hallmarks of cancer (Hanahan and Weinberg, 2011). A study demonstrates that tumor necrosis factor superfamily 15 (TNFSF15) inhibits angiogenesis through IFN-γ-stimulated STAT1 signal (Lu et al., 2014). TNFSF15 functions as a negative modulator of neovascularization by balancing the VEGF/VEGFR ratio (Yang and Li, 2018). Besides, silencing TNFSF15 in the OC ID8 cell inoculation mouse model indeed promotes neovascularization and tumor growth (Deng et al., 2012). Antibody targeting IFN-γ receptor has proved to decrease TNFSF15 expression through IFN-γ/STAT1 signaling. It has been shown that DLX4 promotes proliferation, suppresses apoptosis, and increases microvessel density in OC xenograft models (Trinh et al., 2015). Moreover, DLX4 induces the expression of iNOS by stimulating STAT1 activity, leading to an increase in NO. The major mechanism underlying NO-induced-angiogenesis is the stimulation of VEGF-A (Frank et al., 1999) which can promote angiogenesis and form ascites in OC (Byrne et al., 2003). Furthermore, STAT1 has been proved to suppress neovascularization. For instance, STAT1 upregulates the expression of CXCL9/10/11 that have anti-angiogenesis activities in OC (Weinlander et al., 2008). Another study demonstrates that STAT1 suppresses angiogenesis via inhibiting ITGB1-induced endothelial cell migration (Zhang and Zou, 2015).

### Glycolysis

Some genes in tumorigenesis are associated with glucose metabolism processes including glycolysis, tricarboxylic acid (TCA) cycle, and oxidative phosphorylation. STAT1 is one of such genes to be essential for the promotion of glycolysis (Pitroda et al., 2009) that takes place under oxygen or hypoxia conditions (Akram, 2013). In normal cells, glycolysis is the first step to generate pyruvate in the presence of oxygen, which is oxidized to CO_2_ and H_2_O thereafter (Lunt and Vander Heiden, 2011). On the other hand, cancer cells undergo glycolysis with the increased production of lactate in the presence of oxygen. This interesting phenomenon is termed as Warburg effect or aerobic glycolysis which has been demonstrated in various types of tumors (Lebelo et al., 2019). Cancer cells consume a large amount of intake-glucose through glycolysis in the presence of oxygen. Therefore, glycolysis, also called energy reprogramming, is considered as one of the emerging hallmarks of cancer metabolism (Pavlova and Thompson, 2016) as well as one of the hallmarks of cancer (Hanahan and Weinberg, 2011). A study using the combined transcriptomic-proteomic expression analysis proves that STAT1 promotes the transcription of energy metabolism-associated genes (Pitroda et al., 2009). Among the altered energy metabolism processes, glycolysis shows a significant change of targets both at protein and mRNA levels in response to STAT1 activation. Another study demonstrates that lncRNA ceruloplasmin (NRCP) is associated with cellular metabolism, among which glycolysis is one of the most related metabolic processes (Rupaimoole et al., 2015). NRCP promotes the binding of STAT1 and RNA pol II, leading to an increase in the expression of targeted genes such as glucose-6-phosphate isomerase (GPI). Silencing NRCP results in a significantly decreased expression of GPI, ALDOA, and ALDOC, which further supports the role of the NRCP-STAT1 axis in glycolysis-induced tumorigenesis.

### Immunologic Responsiveness

Signal transducer and activator of transcription 1 enhances anti-tumorigenic immune responses and pro-inflammation function mainly through the induction of cytokines secretion, which in turn leads to an increased amount of anti-tumor immune lymphocytes (Zhang and Liu, 2017). Interestingly, STAT1 increases the expression of interleukin-18 binding protein (IL-18BP) and indoleamine 2,3-dioxygenase (IDO), an immunosuppressive enzyme that suppresses anti-tumor immunity. Evading immune destruction is one of the hallmarks of cancer (Hanahan and Weinberg, 2011). Tumor cells escape immune destruction in different ways, including the alteration of antigens, the decrease of immunogenicity, the changes of the tumor microenvironment (TME), and the decrease of immune responses (Beatty and Gladney, 2015). Generally, STAT1 is identified to be associated with enhanced immune responses through upregulating the amount of cytotoxicity immune cells such as cytotoxic T lymphocytes (CTLs) and natural killer (NK) cells (Meissl et al., 2017; Jin et al., 2019). Contrarily, a microarray analysis proves that STAT1 induces the expression of an immunosuppressive gene *IDO* (Rolvering et al., 2018). The inhibition of phosphorylation and nuclear translocation of STAT1 by bortezomib downregulates IFN-γ-induced IDO expression (Jiang et al., 2017). The *IDO* gene contains one GAS sequence and two IFN-α-stimulated response element (ISRE) domains (Dai and Gupta, 1990). The activation of STAT1 by IFN-γ forms STAT1-dimers and translocated into the nucleus to bind the GAS sequence of IDO and IRF1. The latter further binds to ISRE-1 and ISRE-2, causing an upregulatory effect on IDO expression (Chon et al., 1995). It has been shown that IDO is constitutively expressed in epithelial OC (EOC) cells isolated from ascites and is induced *in vivo* by IL-27 through STAT1 phosphorylation (Carbotti et al., 2015; Petretto et al., 2016). IDO has tryptophan metabolic activity which can transform tryptophan to kynurenine (Munn and Mellor, 2016). The decrease of tryptophan stimulates stress-response kinase GCN2 (Munn et al., 2005), which promotes the differentiation of naïve T cells to T regulatory (Treg) cells (Fallarino et al., 2006). Furthermore, IDO is proved to stimulate the aryl hydrocarbon receptor (AhR), which also promotes the differentiation of T cells to Treg cells (Fallarino et al., 2006; Mezrich et al., 2010) that induce immune tolerance by suppressing cytotoxicity of CTLs (Stein et al., 2011). Hence, STAT1-induced IDO plays a central role in the immune escape of OC cells (de Jong et al., 2011). Besides, the inhibition of IDO suppresses tumor growth by removing the inhibition of NK cells (Mougiakakos et al., 2010), suggesting that IDO is a potential therapeutic target of OC (Wang et al., 2012). Moreover, IDO is related to poor prognosis and paclitaxel resistance in patients with serous-type OC (Okamoto et al., 2005).

Similarly, STAT1 promotes IL-18BP expression, which is an endogenous inhibitor of IL-18, in EOC and primary OC cells (Carbotti et al., 2013). As an immune enhancer, IL-18 promotes anti-malignant tumor immune responses and is generally upregulated in the sera of OC patients (Medina et al., 2014). IL-18BP blocks the function of IL-18 by binding with mat-IL-18. On the other hand, IL-18 stimulates the expression of IL-18BP through IFN-γ and IL-27, leading to negative loop feedback (Veenstra et al., 2002). Thus, the ultimate effects depend on the local concentration ratio of IL-18 and IL-18BP (Carbotti et al., 2013; Medina et al., 2014). Concerning the immune therapy based on IL-18 level in OC patients, IL-18BP is a promising target because it can influence the effects of IL-18 therapy.

Elevated STAT1 increases intra-tumor CD8^+^ T cells, which plays a significant role in tumor inhibition by direct cytotoxicity (Wang et al., 2016; Jifu et al., 2018). Tumor-infiltrating CD8^+^ T cells suppress OC progression and are associated with a favorable prognosis in OC (Preston et al., 2013; Yeung et al., 2018). The Africa American Cancer Epidemiology Study shows that programmed death ligand-1 (PD-L1) positive and IDO positive tumor cells from HGSOC accompany by increased amounts of CD8^+^ T cells (Mills et al., 2019). The upregulated expression of PD-L1 and IDO is induced by phosphorylation of STAT3 and STAT1, respectively (Carbotti et al., 2015). PD-L1 and IDO have been identified to promote the immune escape of cancer cells. The blockage of IDO by iNOS-induced-NO may lead to a favorable prognosis (Munn and Mellor, 2016). Our previous study suggested that the elevated PD-L1 and STAT1 may favor the checkpoint immunotherapy in patients with EOC, but may have a limit in paclitaxel-resistant patients because of the low expression of PD-L1 and STAT1 (Liu et al., 2020).

### Tumor Microenvironment

**FIGURE 3:**
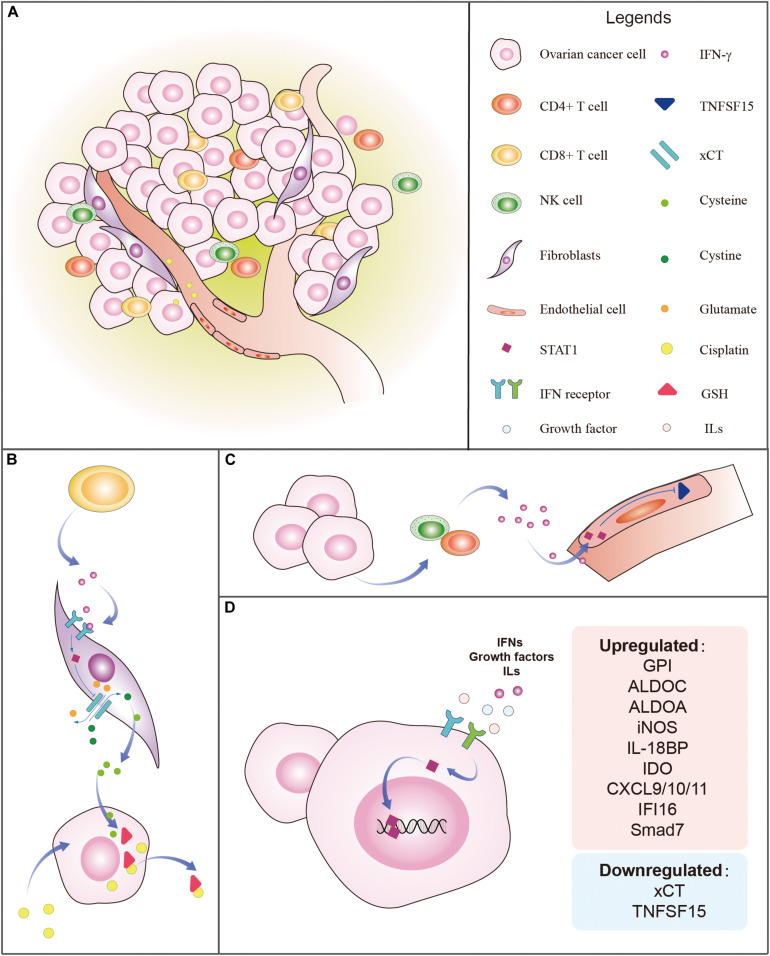
Tumor microenvironment (TME) and regulation of STAT1 in ovarian cancer. **(A)** The typical TME includes ovarian cancer cells, fibroblasts, endothelial cells, vessels, tumor-infiltrating CD4^+^ T cells, CD8^+^ T cells, NK cells, immune regulators, growth factors, and cytokines, etc. **(B)** Example of the influence of the TME on the STAT1 regulatory mechanism. CD8^+^ T cells from the ovarian TME release IFN-γ which binds to IFN-γ receptors to stimulate STAT1. The activated STAT1, in turn, suppresses the expression of xCT which is responsible for the transportation of outer cellular cystine into cells and intracellular glutamate out of the cells. The accumulation of intracellular cystine is then converted into cysteine which is secreted out of fibroblasts. Ovarian cancer intakes cysteine to synthesize GSH which binds to cisplatin to accelerate the efflux of cisplatin. **(C)** Example of the effect of the TME on STAT1 regulatory relationship. Ovarian cancer cells attract tumor-infiltrating CD4^+^ T cells and NK cells that release IFN-γ which activates STAT1 in endothelial cells and suppresses the expression of TNFSF15 that is known to inhibit angiogenesis. **(D)** IFNs, growth factors, and ILs stimulate STAT1 which upregulates and downregulates the targeted genes in ovarian cancer. The genes regulated by STAT1 are listed.

The TME is essential for the secretion of cytokines, growth factors, and immune regulators (Figure 3A). It has been reported that CXCL9/10/11 can promote tissue chemotaxis of CD8^+^ T cells and NK cells by binding to CXCR3, leading to Th1-dominant anti-tumorigenic responses (Rainczuk et al., 2012; Peng et al., 2015). CD8^+^ T cells reduce platinum resistance by secreting IFN-γ, which decreases fibroblast glutathione (GSH) and cysteine metabolism by upregulating glutamyl transferases and suppressing transcription of system x_*c*_-cystine/glutamate antiporter (xCT) via the JAK/STAT1 pathway in OC cells (Wang et al., 2016). GSH induces cisplatin resistance via facilitating cisplatin efflux, preventing cells from oxidative damage and copper (Cu) coupling (Traverso et al., 2013). xCT is responsible for the increase of intracellular cystine. The accumulation of intracellular cystine is converted into cysteine which is secreted out of fibroblasts and then intake by cancer cells to synthesize GSH that binds cisplatin for its efflux (Wang et al., 2018). IFN-γ-stimulated STAT1 binds to the GAS site near the promoter of xCT, blocking the transcription of xCT in fibroblasts within the OC microenvironment (Wang et al., 2016), which binds cisplatin for its efflux (Figure 3B). Immunohistochemical analysis shows that the main sources of IFN-γ are from tumor-infiltrating NK and CD4^+^ T cells (Silva et al., 1994; Bach et al., 1997). IFN-γ then activates STAT1 in endothelial cells and suppresses the expression of TNFSF15 that is known to inhibit angiogenesis (Figure 3C). It is predictable that IFN-γ-stimulated unphosphorylated STAT1 contributes to the downregulation of TNFSF15 in endothelial cells (Lu et al., 2014). IFNs, growth factors, and ILs stimulate STAT1 which upregulates and downregulates the targeted genes in OC (Figure 3D).

### Drug Responsiveness

It has been shown that high levels of STAT1 and STAT1-induced chemokines such as CXCL9/10/11 and CD8A are associated with chemosensitivity in HGSOC (Au et al., 2016). Silenced NRCP can reduce tumor burden and enhance cisplatin sensitivity *in vivo* (Rupaimoole et al., 2015). With a deeper understanding of molecule mechanisms concerning tumorigenesis, anti-tumor activity, tumor microenvironment, and cancer stem cells (CSCs), some mechanisms of drug resistance in OC are emerging (Norouzi-Barough et al., 2018). Typically, drug resistance includes innate and acquired. As to acquired resistance, for example, we can use ABC transporter antibody or artificially pack drugs to avoid being recognized by those ABC transporters. Besides, there are many other measures to cope with drug resistance. It has been proved that STAT1 plays different roles in drug resistance including platinum-, doxorubicin-, and paclitaxel-resistance through transcriptional stimulation of target genes. However, it has a conflicting concern about STAT1-induced drug resistance. For example, interferon-inducible protein 16 (IFI16), which is induced by STAT1, exert anti-tumor effects but protect cancer cells from being killed by chemo-drugs at the same time (Bani et al., 2004). IFI16 is an anti-tumor factor in OC and is also proved to be associated with drug resistance by microarray analysis, which is further verified by immunohistochemistry at the protein level (Ju et al., 2009; Januchowski et al., 2017).

Experiments using OC cell lines *in vitro* come out with different results that iNOS is upregulated in cisplatin-sensitive OV2800 cells. Contrarily, it has been reported that NO induces cisplatin-resistance by increasing levels of glutathione (Turchi, 2006). Concerning the contradictory facts of iNOS and the outcomes of inhibiting iNOS are various (Malone et al., 2006; Kielbik et al., 2019), it is hard to determine whether iNOS is doing good or bad things to OC. Despite the above conflicting results, STAT1 indeed regulates the transcription of iNOS. Although most reports support the idea that OC with high iNOS expression is more sensitive rather than resistant, in general, it is still not clear about iNOS to be considered as a prognosis marker of OC yet (Yu et al., 2017).

### Stemness

Our group is first to demonstrate the involvement of STAT1 in stemness and paclitaxel-resistance in OC cells (Wang et al., 2020). We have found that a clone of paclitaxel-resistant cells shares the characteristics of CSCs and has stemness properties. The stem cell-like paclitaxel-resistant cells have a low expression level of STAT1 and high expression levels of CSC-related markers such as OCT4, CD44, CD133, SOX 2, and NANOG. Moreover, the overexpression of STAT1 in monoclonal paclitaxel-resistant OC cells suppresses the stemness. It is well known that CSCs generally have self-renew and differentiation abilities (Pattabiraman and Weinberg, 2014) and are proved to be associated with drug resistance in OC (Mihanfar et al., 2019). It has also been shown that STAT1 is positively associated with breast CSCs which are characterized by CD24 expression, while CD24 can suppress STAT1 expression (Suyama et al., 2016). It has been reported that CD95 (APO-1/Fas) can maintain stemness in breast cancer cells (Ceppi et al., 2014) and increase stemness by activating STAT1-dependent type I IFN signaling (Qadir et al., 2017). Additionally, the TME plays a significant role in regulating CSCs (Ahmed et al., 2018). Tumor-associated macrophages produce and secret IL-10 which promotes cancer stemness via the JAK/STAT1 and NF-κB/Notch1 pathways in non-small cell lung cancer (Yang et al., 2019). However, whether STAT1 can be a therapeutic target of CSCs is not clear. STAT1-regulating mechanisms in ovarian CSCs remain to explore in the future.

### Crosstalk With the TGF-β Signaling Pathway

The TGF-β signaling pathway is important in regulating cell proliferation, apoptosis, and differentiation and is involved in cellular processes by cross-talking with many other signaling pathways such as epidermal growth factor receptor (EGFR), mitogen-activated protein kinase (MAPK), PI3K/AKT, Notch, Wnt, Hedgehog, and IFN-γ/STAT signaling pathways (Tzavlaki and Moustakas, 2020). For instance, the IFN-γ/STAT1 signaling pathway inhibits TGF-β signaling by upregulating the expression of an inhibitory Smad, Smad7, and then by suppressing TGF-β-induced phosphorylation and nuclear translocation of Smad3 (Ulloa et al., 1999). A previous study also reveals that the TGF-β/Smad3 signaling pathway is enhanced and the production of Smad7 is decreased in STAT1-null mice (Jeong et al., 2006). STAT1 suppresses TGF-β signaling by attenuating the cytoplasmic expression of runt-related transcription factor 2 (Runx2) and blocking the nuclear translocation of Runx2 (Miyazono et al., 2004). A transcriptional activation co-factor p300 is involved in the competition process between IFN-γ/STAT1 and TGF-β/Smad3 signalings in human glomerular endothelial cells (Yang et al., 2005). STAT1 and Smad3 both bind to p300 to enhance the DNA binding ability but IFN-γ attenuates the binding between Smad3 and p300 while TGF-β do not inhibit the binding of STAT1 to p300. This regulatory relationship between STAT1 and TGF-β signaling pathways have been demonstrated in melanoma, ovarian, lung, liver, and prostate cancer cells (Alvarez Mde et al., 2009; Penafuerte et al., 2011; Ayub and Kaul, 2017; Kaowinn et al., 2018; Tian et al., 2018). It has been known that the TGF-β1 signaling pathway has dual effects on tumor growth. TGF-β1 functions as a tumor suppressor inhibiting cell proliferation and stimulate normal cell differentiation at the early stage of cancer (Bierie and Moses, 2006). However, advanced OC cells hijack the TGF-β pathway to avoid an inhibitory effect on tumor cell proliferation. Hence, it is advised that the TGF-β signaling pathway suppresses tumor growth and induces apoptosis at the early stages but promotes metastasis at the advanced stage (Hao et al., 2019). A recent study suggests that TGF-β signaling and STAT1 regulate each other and come into being a negative feedback loop in OC (Tian et al., 2018). Our previous study showed that the overexpression of STAT1 in OC induces cell proliferation through downregulating the TGF-β signaling pathway, whereas TGF-β1 activates the STAT1 pathway by phosphorylating S727 residue of STAT1α which in turn couples with STAT1β and blocks the activation of the TGF-β signaling pathway (Tian et al., 2018). Furthermore, we are first to define the direct interaction of STAT1 with TGF-β receptors physically, which brings the crosstalk at the cell membrane level.

## Clinical Application Potentials

Chemotherapeutic agents such as inhibitors of glycolysis, glucose transporters, hexokinase, pyruvate kinase M2, glutaminase, and isocitrate dehydrogenase have been demonstrated to be effective in cellular experiments and some have already been tested in a clinical trial (Akins et al., 2018). For example, anti-glycolysis agent 3-bromopyruvate selectively targets tumor cells (Gill et al., 2016). As described previously, STAT1 is essential for the promotion of glycolysis; hence, the inhibition of STAT1 might be developed as a strategy for the treatment of OC. Fludarabine has been allowed to treat patients with OC for decades and has been demonstrated to be able to inhibit the phosphorylation of STAT1 (Zhang and Zou, 2015). Bortezomib is a potent and selective proteasome inhibitor and has been used in clinical trials for several cancers. Bortezomib can induce apoptosis and phosphorylate JAK, leading to the phosphorylation and activation of STAT1 (Kao et al., 2013). However, a phase II clinical trial using bortezomib to treat recurrent platinum-sensitive OC shows minimal anti-tumor activity (Aghajanian et al., 2009). Other studies show that the suppression of STAT1 by AG490 significantly inhibits the expression of IDO that avoids immune tolerance (Kobayashi et al., 2015) and induces B-cell chronic lymphocytic leukemia cell apoptosis (Martinez-Lostao et al., 2005). Concerning the role of STAT1 in OC, targeting STAT1 and its-related genes may have clinical potentials.

## Conclusion

Signal transducer and activator of transcription 1 is an important transducer and transcript factor which exerts contradictory effects by directly regulating target genes or cooperating with other transcription factors to mediate tumor progression and drug resistance. STAT1 has a dual role and is involved in the anti-tumor process, tumor progression, drug sensitivity, chemoresistance, and stemness. The crosstalk between the STAT1 signaling pathway and other signaling pathways may enable STAT1 to perform contrary functions during tumorigenesis.

## Future Direction and Perspectives

The specific role of STAT1 in ovarian tumorigenesis is complex. Typically, receptors-associated JAK phosphorylates Y701 of STAT1α/β in response to IFNs and ILs stimulation, whereas the phosphorylation of S727 of STAT1α initiates and executes specific functions of STAT1. Whether STAT1β has an opposite effect of STAT1α on tumorigenesis in OC is still unclear. A previous study reported that STAT1β can block the phosphorylation of STAT1α in B lymphocytes (Baran-Marszak et al., 2004). Therefore, it is predictable that STAT1α performs tumorigenesis function in OC cells while STAT1β balances these malignant-transforming effects of STAT1α in normal cells. Interestingly, TGF-β1 increases the phosphorylation of both Y701 and S727 in normal ovarian surface epithelial cells; while in cancer cells, TGF-β1 only increases S727 phosphorylation but decreases Y701 phosphorylation (Tian et al., 2018). The mechanism for the differential regulation by TGF-β signaling between normal and cancer cells and between Y701 and S727 in OC cells remains unknown and still needs further investigation.

Signal transducer and activator of transcription 1 has been reported to enhance anti-tumor immune response and is considered to inhibit tumor growth (Avalle et al., 2012). However, during tumorigenesis, malignant cells avoid the above impairment through downregulating STAT1 by the methylation of STAT1 in its promoter; the low expression level of STAT1 is observed in a chemoresistant cell line (Wang et al., 2020). Thus, more attention should be paid to the effects of STAT1 on the significance of the TME, the integration of various signals, and the nature of tumor cells. More evidence in the study of stem cell biology is merging but how a drug-resistance is triggered by these cells remains unclear. The role and detailed mechanisms of STAT1 in OC stem cells or the functional process, as well as therapeutic approaches, are needed to explore further.

## Author Contributions

XL and GX were involved in the conception and design of the work and designed the figures. XL performed the literature search, critically analyzed the existing knowledge, and wrote the draft. XL and FW prepared the figures and provided the critical revisions. XX and JZ performed the bibliographic research and provided the critical revisions, also contributed to editing the manuscript. GX contributed to the conception of the work, edited the manuscript, and provided the critical revisions. All authors were involved in manuscript writing, read and approved the final manuscript.

## Conflict of Interest

The authors declare that the research was conducted in the absence of any commercial or financial relationships that could be construed as a potential conflict of interest.

## Abbreviations

ABC: ATP-binding cassette
AEG1: astrocyte-elevated gene-1
AhR: aryl hydrocarbon receptor
AKT: Akt serine/threonine kinase 1
ALDO: aldolase, fructose-bisphosphate
CA: cortistatin A
CCD: coiled-coil domain
CD: cluster of differentiation
CDK: cyclin-dependent kinase
CSC: cancer stem cell
CTLs: cytotoxic T lymphocytes
CXCL: C-X -C motif ligand
CXCR: C-X -C motif receptor
DBD: DNA binding domain
DLX4: distal-less homeobox 4
EGFR: epidermal growth factor receptor
EMT: epithelial to mesenchymal transition
EOC: epithelial ovarian cancer
FAK: focal adhesion kinase
GAS: gamma-interferon activation site
GPI: glucose-6-phosphate isomerase
GSH: glutathione
HGSOC: high-grade serous ovarian cancer
IDO: indoleamine 2,3-dioxygenase
IFI16: interferon-inducible protein 16
IFN: interferon
IFNAR: interferon- α receptor
IFNGR: interferon- γ receptor
IL: interleukin
IL-18BP: interleukin-18 binding protein
iNOS: inducible nitric oxide synthase
IRF: interferon regulatory factor
ISG: interferon-stimulated genes
ISGF: interferon-stimulated gene factor
ISRE: IFN(alpha)-stimulated response element
ITGB1: integrin beta-1
JAK: Janus kinase
LD: linker domain
MAPK: mitogen-activated protein kinase
miR: microRNA
MMP: matrix metallopeptidase
NADH: nicotinamide adenine dinucleotide
ND: N-terminal
NO: nitric oxide
ND: N-terminal
NRCP: lncRNA ceruloplasmin
OCT4: octamer-binding transcription factor 4
OC: ovarian cancer
PARP: poly (ADP-ribose) polymerase
PD-L1: programmed death ligand-1
PI3K: phosphatidylinositol 3-kinase
RNA pol II: RNA polymerase II
Runx2: runt-related transcription factor 2
S727: serine 727 residue
SH2D: Src homology-2 domain
STAT: signal transducer and activator of transcription
TAD: transactivation domain
TCA: tricarboxylic acid
TGF-β: transforming growth factor-β
TME: tumor microenvironment
TNFSF15: tumor necrosis factor superfamily 15
Treg: T regulatory cells
VEGE: vascular endothelial growth factor
xCT: x_*c*_-cystine/glutamate antiporter
Y701: tyrosine 701 residue.
